# Three myths of disseminating COVID-19 information to vulnerable migrants in Japan: lessons learned during the pandemic

**DOI:** 10.1186/s41182-022-00404-9

**Published:** 2022-02-01

**Authors:** Hiroyuki Kiyohara, Yuko Teshima, Haru Angelique Hoshino, Miwa Kanda, Sadatoshi Matsuoka, Azusa Iwamoto, Masami Fujita

**Affiliations:** 1 Bureau of International Health Cooperation, National Center for Global Health and Medicine, 1-21-1, Toyama, Shinjuku-ku, Tokyo, 162-8655 Japan; 2 Department of Community and Global Health, Graduate School of Medicine, The University of Tokyo, 5th Floor, Medical Bldg. 7-3-1 Hongo, Bunkyo-ku, Tokyo, 113-0033 Japan; 3 AIDS Clinical Center, National Center for Global Health and Medicine, 1-21-1, Toyama, Shinjuku-ku, Tokyo, 162-8655 Japan

**Keywords:** COVID-19, Migrants in Japan, Information source, Risk communication, Social media

## Abstract

This paper discusses the challenges of disseminating COVID-19 information to migrant populations by sharing our trial-and-error approach. In 2018, the Migrants’ Neighbor Network & Action (MINNA), a consortium of individuals and organizations that addressed the issues of accessing relevant information and services for migrants in Japan, was launched. Amidst the COVID-19 pandemic, the MINNA attempted to investigate and improve access to health information among Vietnamese, Nepali, and Burmese migrants in Japan. We had three assumptions in distribution of information to reach a large audience, such as building a multilingual website, requesting stakeholders to disseminate information, or posting on Facebook. None of our assumptions were sufficient to reach the target audience in the context of COVID-19, as total number of views that accessed our materials were less than 300 at most. We viewed these myths as the result of overlooking critical elements of effective communication strategies. Eventually, MINNA managed to establish communication with the manager of a Facebook page with the largest number of followers from the Vietnamese community in Japan. Compared with our previous attempts, the messages were delivered to a large audience on the Facebook page, such as the article on COVID-19 vaccines that was viewed more than 300,000 times. In public health emergencies, interactive process of information dissemination is necessary. It is a key component for risk communication and should be prioritized. Breakthroughs in communicating with a larger audience could be possible through partnerships with online communities.

## Purpose

Access to health information is key to achieving better health in the digital age [1]. However, even with accessibility to the Internet, migrants with culturally and linguistically diverse backgrounds tend to be marginalized while accessing health information [2]. Vulnerable migrants were likely excluded from the COVID-19 response information [3]. Therefore, there is an urgent need to include everyone, regardless of their immigration status, during the pandemic [4].

In 2018, the Migrants’ Neighbor Network & Action (MINNA [5]), a consortium of individuals and organizations that addressed the issues of accessing relevant information and services for migrants in Japan, was launched. Amidst the COVID-19 pandemic, the MINNA attempted to investigate and improve access to health information among Vietnamese, Nepali, and Burmese migrants in Japan. This paper discusses the challenges of disseminating information to migrant populations by sharing our trial-and-error approach.

## Demographics of the Vietnamese, Nepali, and Burmese migrants in Japan

As of December 2020, there were almost three million foreign residents in Japan [6]. These three nationalities are some of the fastest-growing foreign communities in Japan (Table 1) [6]. In addition, the young adults, those in their 20s and 30s, constitute a large proportion of the migrants from these communities (Table 2) [6].

**Table 1:** Number of registered foreign residents in Japan by country

	Country and region	Number of residents	Comparison with 5 years ago (%)
1	China	778,112	116.8
2	South Korea	426,908	93.3
**3**	**Vietnam**	**448,053**	**304.9**
4	Philippines​	279,660	121.8
5	Brazil	208,538	120.2
**6**	**Nepal**	**95,982**	**175.2**
7	Indonesia	66,832	186.1
8	Taiwan	55,872	114.7
9	United States	55,761	106.7
10​	Thailand	53,379	117.6
11​	Peru	48,256	101.1
12​	India	38,558	146.9
**13**​	**Myanmar**​	**35,049**	**255.1**
	Total	2,887,116	129.3

Bold indicates the three nationalities, because the three nationalities are the ones we focused in our activities

**Table 2:** Number of Vietnamese, Burmese, and Nepali residents in Japan by age group

Age group	Vietnam (%)	Myanmar (%)	Nepal (%)
–19	7.2	5.0	11.8
20–29	70.9	59.9	45.1
30–39	19.4	24.6	29.4
40–49	16.2	5.6	11.2
50–59	0.5	3.7	2.2
60–	0.3	1.2	0.2

## Outline of the activities

The outline of the activities is illustrated in Table 3.

**Table 3:** Outline of the activities

Activities	2020								2021							
	5	6	7	8	9	10	11	12	1	2	3	4	5	6	7	8
Consultation with individuals and organizations who were supporting vulnerable migrants in Japan	*	*	*	*												
Qualitative research to investigate the barriers and enablers in accessing health-related information					*	*										
Asking key stakeholders to disseminate COVID-19 information to the Vietnamese, Burmese, and Nepali migrants through their Facebook groups								*								
Disseminating information to Vietnamese migrants by posting on large Facebook groups and pages										*						
Disseminating information to Vietnamese migrants in collaboration with the manager of a large Facebook page														*	*	*

Between May and August 2020, we consulted individuals and organizations that supported vulnerable migrants in Japan. It was determined that migrant workers in industries severely impacted by the COVID-19 pandemic were especially facing hardships. These included Vietnamese, Burmese, and Nepali migrants with precarious working conditions, such as language students working part-time, technical interns, and restaurant workers. We qualitatively investigated the barriers and enablers in accessing health-related information among the three nationalities that lived in Japan. (The full report was finalized by the MINNA). In December 2020, in response to the growing number of cases that occurred due to parties and gatherings for Christmas, we asked key stakeholders, including NGOs, health professionals, academic institutions, religious leaders, and migrant community leaders, to disseminate COVID-19 information using their Facebook groups.

In early 2021, we attempted to post COVID-19 information directly on large Facebook groups and pages that focused on Vietnamese individuals, since the Vietnamese New Year, Tet, was in February. Eventually, we collaborated with the manager of a large Facebook page to reach a large number of Vietnamese individuals.

## First myth: It is good enough to provide information on multilingual websites

In Japan, many public entities, such as central and local governments and quasi-governmental agencies, have built multilingual websites for foreign residents. Most of the COVID-19 information is translated into Vietnamese, Burmese, and Nepali languages on these websites.

A number of academic experts and officials suggested that putting COVID-19 related information together on certain multilingual websites should be prioritized to disseminate information widely to non-Japanese speakers. However, according to interviews with migrants from the abovementioned three countries, these websites are hardly accessed and Facebook acts as an almost exclusive source of information. These individuals obtained their COVID-19 information by browsing posts and comments on Facebook groups and pages [7].

## Second myth: It is good enough to ask key stakeholders to disseminate information through Facebook groups

After the first myth was debunked, we assumed that information would be better conveyed by Facebook groups run by key stakeholders. Some key stakeholders created small Facebook groups, where migrants got acquainted with and mutually supported each other.

**Fig. 1:**
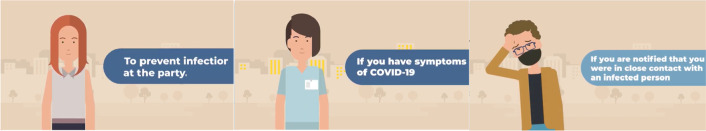
YouTube videos created by our group (MINNA) (Left: to prevent infections at a gathering, Center: if you have symptoms of COVID-19, and Right: if you are notified that you were in close contact with an infected person)

Another assumption was that information would be better transferred through visual materials rather than text messages. A total of three videos were created in five languages (Simple Japanese, English, Vietnamese, Nepali, and Burmese) and uploaded to YouTube in December 2020 to raise awareness regarding COVID-19 prevention (Fig. 1). The video contents were reviewed and approved by an expert panel that consisted of health professionals and other experts who support the migrant community.

MINNA asked 21 key stakeholders to disseminate YouTube videos through Facebook groups. Table 4 shows the topics of each video as well as the number of views 20 days after being shared by key stakeholders. Contrary to our assumptions, the number of views was less than 300, which indicated that the dissemination effect was small.

**Table 4:** Numbers of views of three videos in five languages

Title of the video	Number of views for each language				
	Simple Japanese	English	Vietnamese	Nepali	Burmese
To prevent infections at a gathering	102	231	213	175	157
If you have symptoms of COVID-19	53	91	54	81	61
If you are notified that you were in close contact with an infected person	80	155	41	172	58

The figures above are the number of views of YouTube video within 20 days after being released

## Third myth: It is good enough to post on large Facebook groups or pages

Apart from Facebook groups run by key stakeholders, we recognized the presence of large Facebook groups and pages in each migrant community. It was found that most of these groups and pages were not about nourishing migrant communities through mutual aid but aimed to share daily matters among the mass population, such as entertainment, language learning, and job searches. We assumed that posting on large Facebook groups/pages would enable us to reach a large audience.

A keyword search was conducted to identify large Facebook groups and pages (Table 5). Our results are presented in Table 5. We joined three Facebook groups and posted articles on their timelines. However, one group deleted our posts. The remaining two groups had more than 570,000 combined members who could potentially view our videos. However, the total number of views for 10 days was 80, which overturned our assumption.

**Table 5:** List of Facebook groups and pages for Vietnamese people living in Japan

No.	Type	Number of followers/members as of December 2020	Contact method	Outcomes of contact
1	Page A	809,000	Directly contacted a manager	Received a reply from a manager
2	Page B	729,000	Directly contacted a manager	No reply
3	Group A	312,000	Requested to join a group	Accepted
4	Group B	264,000	Requested to join a group	Accepted
5	Group C	248,000	Requested to join a group	Accepted, but the video links were deleted later by a group manager
6	Group D	184,000	Requested to join a group	Request was not accepted
7	Group E	155,000	Requested to join a group	Request was not accepted
8	Page C	152,000	Directly contacted a manager	No reply
9	Page D	149,000	Directly contacted a manager	No reply
10	Group F	143,000	Requested to join a group	Request was not accepted

Facebook pages are visible to everyone, and the authors were able to directly contact each manager of the page. Facebook groups, on the other hand, are only for those who are allowed to join the group. Hence, the authors had to request to join a group to post on the timeline

## Silver lining after the three myths were debunked

Finally, MINNA managed to establish communication with the manager of a Facebook page with the largest number of followers from the Vietnamese community in Japan.

**Fig. 2:**
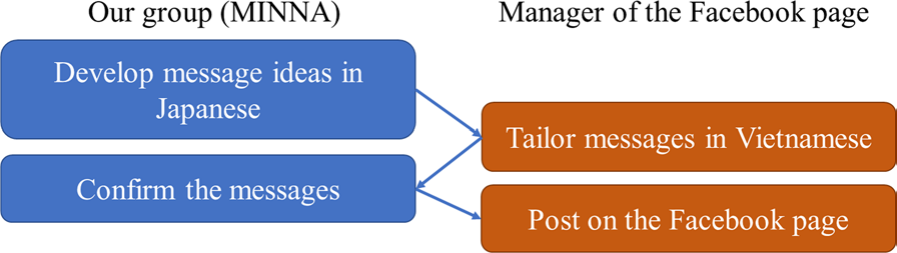
Flow of developing Vietnamese messages on Facebook

Figure 2 describes the flow of developing messages until they were posted on Facebook. MINNA chose four topics regarding COVID-19 (Table 6) and developed the message ideas in Japanese. The manager modified our messages in Vietnamese to better understand the interests and needs of the audience. The messages were finalized with MINNA and posted on the Facebook page. Compared with our previous attempts, these messages were delivered to a large audience. For instance, the article on COVID-19 vaccines was viewed more than 300,000 times (Table 6). We acknowledge that the viewers may have not been limited to Vietnamese migrants living in Japan, in fact some might have been viewed from Vietnam. However, it is safe to deduce that our messages reached a substantial proportion of approximately 440,000 Vietnamese individuals living in Japan [6].

**Table 6:** Total number of views, engagements, shares, and comment

Post themes	Total number of views	Total number of engagements	Total number of shares	Total number of comments
COVID-19 variants	94,000	3264	196	250
Prevention and wearing face masks	41,000	655	9	32
COVID-19 vaccines	303,000	3407	286	384
Flowchart for the management of COVID-19 patients in Japan	155,000	2153	288	72

## Discussion

We highlighted three myths in disseminating information to culturally and linguistically diverse migrants. None of our assumptions were sufficient to reach the target audience in the context of COVID-19. We viewed these myths as the result of overlooking critical elements of effective communication strategies.

The rise of social media has amplified variations in the sources of health information. Our first finding that migrants hardly accessed public multilingual websites and relied on Facebook as a source of COVID-19 information was consistent with previous research. According to Ali et al., social media was the most utilized source for COVID-19 information, followed by the World Health Organization. However, public entity websites were not mentioned in this study [8]. In general, multilingual websites are a welcome support for migrants, but they are not necessarily the most accessed platforms. The importance of identifying a suitable information channel should not be disregarded [9, 10]. Otherwise, information may not be widely accessible despite the efforts undertaken.

Traditionally, information is delivered one-way, from experts to uninformed nonexperts [11]. In public health emergencies, the circumstances are fluid and information is regularly updated. In such unstable situations, the importance of risk communication, defined as “an interactive process of exchange of information and opinion between risk assessors, risk managers, and other interested parties,” [11] cannot be overstated. The interactive process is a core component of risk communication. However, we simply asked key stakeholders to share the video links, which lacked the interactions required for risk communication.

The technological nature of Facebook, which secures high privacy for its users, daunted us when we attempted to use large Facebook groups and pages, as most of them restricted others from posting on their timelines freely. Even if we were approved to join, our posts were soon replaced by newer posts, which were constantly added, and hence, they did not remain at the top of the page for long. As a result, despite a large population of potential viewers, only a few individuals clicked on our video links and viewed them. This suggests that when using a large Facebook group or page as a communication channel, it is important to build a good partnership with the managers who control the posts and timelines.

It is noteworthy that we forged a partnership with the manager of a large Facebook page with more than 800,000 followers. The manager founded his own corporate to run the Facebook page, where health information was just one topic among others, such as posts related to daily news, sports, and entertainment. When we first spoke to the manager, we clarified our goal of delivering credible information to the Vietnamese community in Japan. The manager said, “This collaboration is not for profit but for Vietnamese people in Japan, which I think is very worthwhile. I can accept your request.” Honesty and being public-spirited might have promoted this collaboration. As shown in Fig. 2, the messages were developed by a combined effort from both MINNA and the manager of the Facebook page. The manager did not translate Japanese into Vietnamese verbatim but tailored our messages to the Vietnamese audience. Tailoring messages for audiences is recommended in emergency risk communication [12] and is an essential attribute for knowledge to be transferred [13].

Although our findings are suggestive, they must be interpreted with caution. Our approach of building a partnership with an online community manager may not be generalizable to other migrant subgroups. Despite ubiquitous Internet use throughout Japan, it remains to be seen whether such large Facebook pages can enable the engagement of low-skilled laborers and undocumented migrants who cannot afford data roaming. The MINNA will continue a partnership with the manager and explore a better way to empower the Vietnamese community in Japan. Furthermore, we will seek an opportunity to expand our scope for cooperation with other migrant groups, such as the Burmese and Nepali migrants in Japan.

## Conclusion

As of October 2021, COVID-19 is still an ongoing public health problem, affecting people globally. Vulnerable migrants, especially those with culturally and linguistically diverse backgrounds, should not be marginalized regarding information access during a health crisis. Information dissemination is not a linear process, such as simply building a multilingual website, asking stakeholders to disseminate information, or posting on Facebook. The interactive process of information dissemination, which is a component of risk communication, should be highlighted. Breakthroughs in communicating with a larger audience could be possible through partnerships with online communities.

## Data Availability

The data set used during the current study is available from the corresponding author on reasonable request.

## Abbreviations

COVID-19: Coronavirus disease 2019
MINNA: Migrants’ Neighbor Network & Action
